# Supplementary material for: Tead1a Initiates Transcriptional Priming Through the TEAD1a/YAP‐Notch1‐Spi1/Cebpα Axis to Promote Neutrophil Fate
Source: Adv Sci (Weinh). 2025 Aug 28;12(41):e05441. doi: 10.1002/advs.202505441 (PMC12591127; doi:10.1002/advs.202505441)
Supplement: Supplementary file 4 — Supplementary Dataset [file ADVS-12-e05441-s004.zip › 3-2.zip!/3-2/02-2.cluster/cluster3/Enrich/ego_BP.pdf]

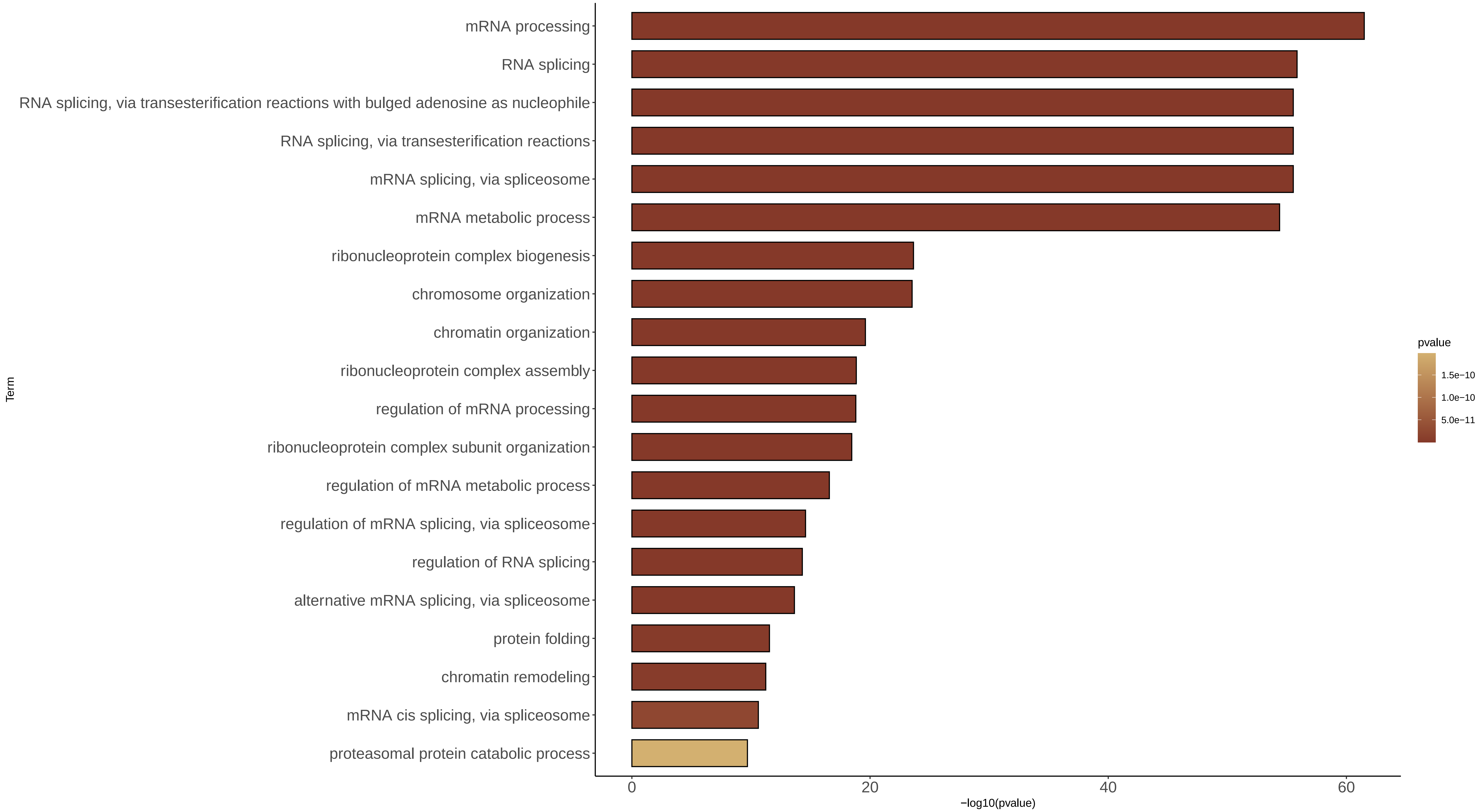
mRNA processing
RNA splicing
RNA splicing, via transesterification reactions with bulged adenosine as nucleophile
RNA splicing, via transesterification reactions
mRNA splicing, via spliceosome
mRNA metabolic process
ribonucleoprotein complex biogenesis
chromosome organization
chromatin organization
ribonucleoprotein complex assembly
regulation of mRNA processing
ribonucleoprotein complex subunit organization
regulation of mRNA metabolic process
regulation of mRNA splicing, via spliceosome
regulation of RNA splicing
alternative mRNA splicing, via spliceosome
protein folding
chromatin remodeling
mRNA cis splicing, via spliceosome
proteasomal protein catabolic process
Term
0
20
40
60
−log10(pvalue)
pvalue
1.5e−10
1.0e−10
5.0e−11
